# Adolescents’ Concerns About School Violence or Shootings and Association With Depressive, Anxiety, and Panic Symptoms

**DOI:** 10.1001/jamanetworkopen.2021.32131

**Published:** 2021-11-01

**Authors:** Kira E. Riehm, Ramin Mojtabai, Leslie B. Adams, Evan A. Krueger, Delvon T. Mattingly, Paul S. Nestadt, Adam M. Leventhal

**Affiliations:** 1 Department of Mental Health, Bloomberg School of Public Health, The Johns Hopkins University, Baltimore, Maryland; 2 Department of Preventive Medicine, Keck School of Medicine, University of Southern California, Los Angeles; 3 Center for Social Epidemiology and Population Health, Department of Epidemiology, University of Michigan School of Public Health, Ann Arbor; 4 Department of Psychiatry and Behavioral Sciences, Johns Hopkins School of Medicine, Baltimore, Maryland; 5 Institute for Addiction Science, Keck School of Medicine, University of Southern California, Los Angeles; 6 Department of Psychology, University of Southern California, Los Angeles

## Abstract

**Importance:**

The prevalence of internalizing problems among US adolescents has risen in the past decade. The extent to which concerns about school violence or shootings are associated with risk of internalizing problems is unknown.

**Objective:**

To examine the prospective association of concern, worry, and stress related to school violence or shootings with internalizing problems and to examine sex and racial and ethnic differences in the magnitude of the associations.

**Design, Setting, and Participants:**

This longitudinal cohort study involved 3 surveys administered 6 months apart (fall of grade 11 [prebaseline]; spring of grade 11 [baseline]; and fall of grade 12 [follow-up]) from 2015 to 2016. Participants included 2263 students from 10 high schools in Los Angeles, California. Analyses were performed from April 29, 2020, to April 8, 2021.

**Exposures:**

Baseline self-reported level of concern, worry, and stress about shootings or violence at the student’s school or other schools, each rated on 5-point scales (ranging from not at all [0] to extremely [4]) with a mean score calculated as a 3-item composite index rescaled into *z*-score standard deviation units.

**Main Outcomes and Measures:**

Surpassing clinically significant or borderline significant thresholds for major depressive disorder, generalized anxiety disorder, or panic disorder based on symptom ratings on the Revised Children’s Anxiety and Depression Scale at 6-month follow-up.

**Results:**

Of the 2263 students included in the analyses (1250 [55.2%] girls; mean [SD] age, 16.5 [0.4] years), appreciable proportions reported being very or extremely concerned (850 0f 2226 [38.2%]), worried (703 of 2209 [31.8%]), or stressed (332 of 2183 [15.2%]) about shootings or violence at their school or other schools. After adjusting for prebaseline covariates, concerns about school violence or shootings were associated with clinically significant generalized anxiety symptoms (odds ratio [OR], 1.31; 95% CI, 1.15-1.50) and panic symptoms (OR, 1.18; 95% CI, 1.05-1.32), but not depressive symptoms (OR, 1.13; 95% CI, 0.99-1.30) at the 6-month follow-up. There was a significant association between concern with school violence or shootings and depressive symptoms for Black youth (OR, 3.15; 95% CI, 1.38-7.19) and non-Hispanic/Latinx White youth (OR, 1.62 [95% CI, 1.25-2.09]) but not for youth of other races and ethnicities (OR for Asian, 1.26 [95% CI, 0.86-1.85]; OR for Hispanic/Latinx, 0.94 [95% CI, 0.76-1.16]; OR for other, 0.93 [95% CI, 0.54-1.61]). Sex did not moderate these associations.

**Conclusions and Relevance:**

The findings of this study suggest that concern, worry, and stress related to school violence or shootings may be risk factors for internalizing problems among adolescents, with variation in the strength of the association by race/ethnicity.

## Key Points

**Question:**

Is reported concern about school violence or shootings associated with internalizing problems among adolescents?

**Findings:**

In a cohort study of 2263 adolescents, greater concern about school violence or shootings was prospectively associated with increased odds of reporting generalized anxiety and panic symptoms 6 months later. There was no overall association between concern about school violence and depressive symptoms.

**Meaning:**

These findings suggest that concerns about school violence or shootings might be a risk factor for internalizing problems among adolescents that may warrant public health attention.

## Teaser

This cohort study examines the association between concern, worry, and stress about school shootings and depression, generalized anxiety disorder, or panic disorder among US adolescents.

## Introduction

Since approximately 2011, the prevalence of major depressive disorder and depressive symptoms has increased among US adolescents.^1,2,3^ As of 2014, the 12-month prevalence of major depressive episodes was 11.3%, representing a significant increase from 8.7% in 2005.^1^ In 2018, 70% of youth reported anxiety and depression (collectively termed *internalizing problems*)^4^ to be major problems among their peers.^5^ Given the strong association between adolescent internalizing problems and subsequent adverse outcomes in adulthood,^6^ research is needed to identify factors with a high exposure prevalence that increase the risk of internalizing problems among adolescents and are contemporarily salient.

Explanations for increasing internalizing problem trends are largely speculative, and some hypotheses include increases in digital media use, exposure to cyberbullying, economic recessions, decreased sleep quantity and quality, concerns about the environment, and exposure to the consequences of the opioid epidemic.^2,3,7^ An emerging and increasingly salient stressor for youth is exposure to violence in school settings and concern about school shootings. In 2019, approximately 8.7% of students from 9th to 12th grade reported that they did not attend school owing to safety concerns at least once in the past 30 days—an increase of more than 2-fold since 1993.^8^ School shootings are often widely publicized, and mere knowledge about such shootings might influence youths’ perceptions of safety at their own school, even if gun violence in their own schools and communities has never occurred.^9^ For adolescent residents of communities affected by gun violence, the portrayal of school shootings in the media could be particularly distressing.

In 2018, more than half of US students reported feeling somewhat or very worried about the possibility of a shooting happening at their school.^10^ Of note, these worries were more common among female, Black, and Hispanic students^10^ and may contribute to disparities in adolescent mental health. Collectively, these findings raise the possibility that youths’ concern about violence and school shootings may be a contributing factor to internalizing problems and may differentially relate to internalizing problems by sex, race, and ethnicity.

In this longitudinal cohort study, we tested the hypothesis that adolescents who reported greater concern, worry, and stress associated with school violence or shootings would report greater odds of depressive, generalized anxiety, and panic symptoms at a 6-month follow-up. We also examined whether these associations differed by sex, race, and ethnicity. To our knowledge, this is the first study to examine the association between concern about school shootings and internalizing problems among US adolescents.

## Methods

### Participants

The data in this cohort study were drawn from the Happiness and Health Study, a longitudinal cohort of adolescents from 10 public high schools in Los Angeles, California.^11^ Participants who were in the 9th grade and their parents provided active assent and consent before enrolling in the study. Thereafter, assessments were administered every 6 months in classrooms. Students who were not present on the day of survey administration completed abbreviated surveys that omitted key measures and were therefore not included in this study. The University of Southern California institutional review board approved the study. This study followed the Strengthening the Reporting of Observational Studies in Epidemiology (STROBE) reporting guidelines.

Questions regarding concern with school violence or shootings were first introduced during the 11th grade survey assessment in spring of 2016, which represents the baseline wave for the current study; surveys 6 months before (fall of 2015 in 11th grade) and 6 months after (fall of 2016 in 12th grade) represent the prebaseline and follow-up waves, respectively. The sample included students who completed assessments at all 3 waves; had complete data for sex, race, and ethnicity; and had measurements for the concern about school violence or shootings scale and at least 1 of the internalizing problem outcomes. A participant flow diagram is presented in the eFigure in the Supplement. Of 4100 eligible 9th grade students, 3396 enrolled in the study, and data from 2263 participants were included in analyses. eTable 1 in the Supplement displays demographic comparisons between participants included in analyses and those excluded from the sample.

### Measures

#### Baseline Concern, Worry, and Stress About School Violence or Shootings

In a survey section labeled “Issues in Society,” students were asked to rate their degree of “concern, worry, and stress towards the following issues in terms of their effect on [them] personally” (the eMethods in the Supplement provides a complete list of survey items). Three separate items measured the degree to which adolescents were concerned, worried, and stressed with regard to “shootings or violence at your school or other schools,” with response options of not at all (0), slightly (1), somewhat (2), very (3), and extremely (4). As in prior studies using similar measures of concern for other issues,^12^ we formed a composite index reflecting the level of concern about school violence or shootings by calculating the mean of the 3 items (range, 0-4). Composite scores are hereinafter referred to as concerns about school violence or shootings. This measure had high internal consistency as measured by both the Cronbach α (0.90) and item-total correlations (concern: 0.82, worry: 0.88, stress: 0.74). To assist with interpretability, we converted scores for this measure into SD unit *z* scores for regression models.

#### Internalizing Problems at Follow-up

The outcomes of interest included symptom ratings for major depressive disorder, generalized anxiety disorder, and panic disorder that surpassed the borderline clinically significant or clinically significant thresholds. These were measured using the respective subscales of the 47-item Revised Children’s Anxiety and Depression Scale (RCADS), which consisted of 10 items for major depressive disorder, 6 items for generalized anxiety disorder, and 9 items for panic disorder. Adolescents were asked to indicate how often certain things happened to them (eg, “I feel sad or empty,” “I worry that bad things will happen to me,” “When I have a problem, I feel shaky”). Response options were never (0), sometimes (1), often (2), or always (3). Total scores for each disorder were obtained by summing the item scores. The RCADS has age- and sex-specific normative samples in which symptom scores can be identified as borderline clinically significant (T score of 65-69) or clinically significant (T score of ≥70). We used these to create a binary outcome variable for each disorder (does vs does not surpass borderline or clinical threshold). The RCADS has been extensively validated in both clinical and school-based samples and has been found to accurately distinguish between diagnostic groups identified by semistructured interviews.^13,14^

#### Prebaseline Covariates

Covariates were selected on the basis of existing literature on the association between perceived school safety and mental health.^15,16^ Covariates were assessed at a time before the primary regressor variable.^17^ For sociodemographic characteristics, we included age, sex (female or male), race and ethnicity (Asian, Black, Hispanic/Latinx, non-Hispanic/Latinx White, or other [American Indian or Alaska Native, Native Hawaiian or Pacific Islander, multiracial, and other race or ethnicity]) and eligibility for free or reduced-cost lunch (not eligible or eligible). Externalizing, disruptive behavioral problems included attention-deficit/hyperactivity symptoms, measured with the Current Symptoms Self-report Form,^18^ and delinquent behavior, measured with a scale assessing the frequency of 11 behaviors in the past 6 months (eg, lying to parents, skipping school),^19^ past 6-month use of alcohol (yes or no), and past 6-month use of nicotine, marijuana, and/or illicit drugs (yes or no). School-related covariates included past 30-day exposure to cyberbullying, measured with the 8-item Cyberbullying and Online Aggression Survey Instrument^20^ (summary scores dichotomized into yes or no), and subjective social status at school, measured with the MacArthur Scale of Subjective Social Status (scores range from 1-10, with higher scores indicating greater perceived status).^21^ Finally, we also adjusted for continuous scores from the prebaseline major depressive disorder, generalized anxiety disorder, and panic disorder subscales of the RCADS (described above).

### Statistical Analysis

#### Association of Concern About School Violence or Shootings With Internalizing Problems

Logistic regression was used to estimate the association of concern about school violence or shootings with depressive, generalized anxiety, and panic symptoms. Regression coefficients were exponentiated for interpretation as odds ratios (ORs) with associated 95% CIs; for the composite concern index, these represent the change in odds of reporting a borderline or clinically significant symptom per 1-SD increase in concern. We estimated unadjusted and adjusted models for each internalizing problem; the adjusted models included all 13 covariates listed above.

#### Moderation by Sex, Race, and Ethnicity

We examined whether the associations between concern about school violence or shootings with each outcome differed according to sex and to race and ethnicity by estimating separate adjusted models. These included omnibus interaction terms between concern about school violence or shootings and sex or all race and ethnicity categories.

#### Sensitivity Analyses and Missing Data

Sensitivity analyses, which were conducted to test modeling decisions and other assumptions, are described in the eMethods in the Supplement. To account for missing data on covariates in our sample, we performed multiple imputation using chained equations^22^ and generated 15 imputed data sets. Huber-White robust SEs were calculated to account for clustering by school.^23^ Statistical significance was assessed at 2-sided *P* < .05. All analyses were conducted using RStudio, version 1.2.5042, and R, version 4.0.0 (R Program for Statistical Computing). Analyses were preformed from April 29, 2020, to April 8, 2021.

## Results

### Descriptive Results

Among the 2263 students included in the analytic sample, the mean (SD) age at the prebaseline survey was 16.5 (0.4) years; 1250 students (55.2%) were girls and 1013 students (44.8%) were boys. The sample was racially and ethnically diverse; 444 students (19.6%) were Asian, 89 (3.9%) were Black, 1001 (44.2%) were Hispanic/Latinx, 375 (16.6%) were non-Hispanic/Latinx White, and 354 (15.6%) reported another race or ethnicity. Complete descriptive statistics are reported in Table 1.

**Table 1.** Descriptive Characteristics for Analytic Sample at the Prebaseline Wave

Characteristic	Data^a^
Sex	
Female	1250 (55.2)
Male	1013 (44.8)
Age, mean (SD), y	16.5 (0.4)
Race and ethnicity	
Asian	444 (19.6)
Black	89 (3.9)
Hispanic/Latinx	1001 (44.2)
Non-Hispanic/Latinx White	375 (16.6)
Other^b^	354 (15.6)
Free or reduced-cost lunch	
Not eligible	1148 (53.6)
Eligible	992 (46.4)
Cyberbullying	
Not bullied	1896 (88.7)
Bullied	242 (11.3)
Subjective social status at school, mean (SD) score^c^	6.8 (1.8)
Delinquent behavior, mean (SD) score^d^	13.8 (4.4)
ADHD symptoms, mean (SD) score^e^	11.0 (9.7)
Past 6-mo alcohol use	
No	1639 (73.7)
Yes	584 (26.3)
Past 6-mo nicotine, cannabis, and/or illicit drug use	
No	1716 (76.9)
Yes	514 (23.0)

Abbreviation: ADHD, attention-deficit/hyperactivity.

^a^ Unless otherwise indicated, data are expressed as number (%) of students. Percentages have been rounded and may not total 100. Descriptive statistics are based on available data and numbers may total less than the 2263 students included in the analysis.

^b^ Includes American Indian or Alaska Native, Native Hawaiian or Pacific Islander, multiracial, and other race or ethnicity.

^c^ Measured with the MacArthur Scale of Subjective Social Status (range, 1-10, with higher scores indicating greater perceived status).

^d^ Measured with a scale assessing the frequency of 11 behaviors (eg, lying to parents, skipping school) in the past 6 months (range, 6-66; higher scores indicate engaging in delinquent behaviors).

^e^ Measured with the 18-item Current Symptoms Self-report Form; each item is scored from 0 to 3, and a total score is obtained by summing the items (range, 0-54, with higher scores indicating a higher frequency of experiencing symptoms of ADHD).

Table 2 shows the proportion of students selecting each response option for the survey items assessing concern, worry, and stress about school violence or shootings. A considerable proportion of students reported being very or extremely concerned (850 of 2226 [38.2%]), worried (703 of 2209 [31.8%]), or stressed (332 of 2183 [15.2%]) about shootings or violence at their school or other schools. Girls were significantly more likely than boys to report higher levels of concern (very or extremely, 556 of 1231 [45.2%] vs 294 of 1309 [22.5%]), worry (very or extremely, 473 of 1228 [38.5%] vs of 230 of 981 [23.4%]), and stress (very or extremely, 240 of 1209 [19.9%] vs 92 of 974 [9.4%]), as well as higher mean (SD) composite scores (1.84 [1.27] vs 1.27 [1.20]; *P* < .001 for all). No significant differences were observed by race/ethnicity in concern, worry, stress, or composite scores.

**Table 2.** Descriptive Statistics of Baseline Concern, Worry, and Stress About School Violence or Shootings, in the Complete Sample and Stratified by Sex, Race, and Ethnicity^a^

School violence or shootings item	Complete sample	Stratified by student sex			Stratified by student race and ethnicity					
		Female	Male	*P* value^b^	Asian	Black	Hispanic/Latinx	Non-Hispanic/Latinx White	Other^c^	*P* value^b^
Concern										
Not at all	521 (23.4)	207 (16.8)	314 (31.5)	<.001	93 (25.1)	30 (34.1)	230 (23.3)	87 (20.1)	81 (23.1)	.06
Slightly	348 (15.6)	183 (14.9)	165 (16.6)		74 (20.0)	16 (18.2)	144 (14.6)	63 (14.6)	51 (14.6)	
Somewhat	507 (22.8)	285 (23.2)	222 (22.3)		72 (19.5)	12 (13.6)	228 (23.1)	116 (26.9)	79 (22.6)	
Very	432 (19.4)	272 (22.1)	160 (16.1)		72 (19.5)	14 (15.9)	184 (18.7)	87 (20.1)	75 (21.4)	
Extremely	418 (18.8)	284 (23.1)	134 (13.5)		59 (15.9)	16 (18.2)	200 (20.3)	79 (18.3)	64 (18.3)	
Worry										
Not at all	625 (28.3)	242 (19.7)	383 (39.0)	<.001	111 (30.1)	33 (37.5)	261 (26.9)	111 (25.6)	109 (31.4)	.17
Slightly	399 (18.1)	222 (18.1)	177 (18.0)		84 (22.8)	14 (15.9)	170 (17.5)	75 (17.3)	56 (16.1)	
Somewhat	482 (21.8)	291 (23.7)	191 (19.5)		67 (18.2)	14 (15.9)	227 (23.4)	105 (24.2)	69 (19.9)	
Very	346 (15.7)	225 (18.3)	121 (12.3)		53 (14.4)	11 (12.5)	152 (15.6)	75 (17.3)	55 (15.9)	
Extremely	357 (16.2)	248 (20.2)	109 (11.1)		54 (14.6)	16 (18.2)	162 (16.7)	67 (15.5)	58 (16.7)	
Stress										
Not at all	1092 (50.0)	489 (40.4)	603 (61.9)	<.001	197 (53.8)	53 (61.6)	471 (49.4)	198 (45.9)	173 (50.0)	.13
Slightly	417 (19.1)	271 (22.4)	146 (15.0)		66 (18.0)	9 (10.5)	184 (19.3)	92 (21.3)	66 (19.1)	
Somewhat	342 (15.7)	209 (17.3)	133 (13.7)		49 (13.4)	12 (14.0)	142 (14.9)	80 (18.6)	59 (17.1)	
Very	175 (8.0)	124 (10.3)	51 (5.2)		28 (7.7)	2 (2.3)	84 (8.8)	33 (7.7)	28 (8.1)	
Extremely	157 (7.2)	116 (9.6)	41 (4.2)		26 (7.1)	10 (11.6)	73 (7.7)	28 (6.5)	20 (5.8)	
Composite score, mean (SD)	1.58 (1.27)	1.84 (1.27)	1.27 (1.20)	**<** .001	1.46 (1.27)	1.38 (1.37)	1.63 (1.29)	1.64 (1.22)	1.57 (1.27)	.10

^a^ Unless otherwise specified, data are presented as number (%) of students. Estimates are based on available data for each item.

^b^ Calculated from χ^2^ tests for between-group differences for each item or from analysis of variance tests for the composite scores.

^c^ Includes American Indian or Alaska Native, Native Hawaiian or Pacific Islander, multiracial, and other race or ethnicity.

At follow-up, the proportion of adolescents surpassing the borderline/clinically significant threshold was 350 of 2204 (15.9%) for major depressive disorder, 273 of 2205 (12.4%) for generalized anxiety disorder, and 302 of 2202 (13.7%) for panic disorder. eTable 2 in the Supplement shows the proportions of adolescents surpassing the borderline/clinically significant thresholds for each internalizing problem at follow-up, stratified by their status at the prebaseline wave.

### Association of Concerns About School Violence or Shootings With Internalizing Problems

In unadjusted analyses, concern about school violence or shootings at baseline was associated with surpassing the borderline/clinically significant threshold for each internalizing problem (OR for major depressive disorder, 1.19 [95% CI, 1.06-1.33]; OR for generalized anxiety disorder, 1.48 [95% CI, 1.34-1.65]; OR for panic disorder 1.20 [95% CI, 1.08-1.33]). These ORs indicated that each 1-SD unit increase in level of concern about shootings was associated with 19% greater odds of surpassing the symptom level clinical/borderline threshold for major depressive disorder, 48% for generalized anxiety disorder, and 20% for panic disorder. In adjusted analyses, which included adjustment for various mental health problems such as internalizing symptoms, concern about school violence or shootings was associated with surpassing the borderline/clinically significant threshold for generalized anxiety disorder (OR, 1.31; 95% CI, 1.15-1.50) and panic disorder (OR, 1.18; 95% CI, 1.05-1.32), but not major depressive disorder (OR, 1.13; 95% CI, 0.99-1.30). Complete results, including covariate estimates, are shown in Table 3.

**Table 3.** Unadjusted and Adjusted Association Between Concern About School Violence or Shootings and Depressive, Generalized Anxiety, and Panic Symptoms

Regressor variable	Symptom outcome at 6-mo follow-up, OR (95% CI)		
	General depressive disorder	Generalized anxiety disorder	Panic disorder
Unadjusted model			
Concern about school violence or shootings^a^	1.19 (1.06-1.33)	1.48 (1.34-1.65)	1.20 (1.08-1.33)
Adjusted model			
Concern about school violence or shootings^a^	1.13 (0.99-1.30)	1.31 (1.15-1.50)	1.18 (1.05-1.32)
Male vs female sex	1.54 (1.24-1.93)	1.60 (1.19-2.15)	2.60 (1.88-3.58)
Age, y^b^	1.13 (0.78-1.63)	0.78 (0.59-1.04)	1.10 (0.67-1.82)
Race and ethnicity			
Asian	1.33 (0.94-1.88)	1.14 (0.49-2.67)	1.02 (0.56-1.88)
Black	1.18 (0.77-1.82)	1.03 (0.42-2.55)	1.24 (0.62-2.48)
Hispanic/Latinx	1.05 (0.81-1.38)	1.37 (0.87-2.15)	0.76 (0.50-1.15)
Non-Hispanic/Latinx White	1 [Reference]	1 [Reference]	1 [Reference]
Other^c^	1.44 (0.93-2.23)	1.26 (0.57-2.78)	0.96 (0.69-1.32)
Eligible for free or reduced cost lunch vs not eligible^b^	1.25 (0.88-1.78)	1.02 (0.80-1.29)	1.26 (0.89-1.80)
Exposure to cyberbullying vs none^b^	1.14 (0.72-1.80)	1.04 (0.65-1.67)	1.32 (0.92-1.89)
Subjective social status at school^b^	0.93 (0.86-1.01)	0.98 (0.87-1.11)	1.02 (0.93-1.11)
Delinquent behavior^b^	1.00 (0.96-1.04)	1.03 (1.01-1.04)	0.97 (0.92-1.02)
ADHD symptoms^b^	1.03 (1.02-1.05)	1.02 (1.00-1.04)	1.04 (1.03-1.06)
Past 6-mo alcohol use vs no use^b^	0.95 (0.64-1.41)	0.91 0.64-1.30)	0.90 (0.65-1.25)
Past 6-mo use of nicotine, cannabis, and/or illicit drugs vs no use^b^	0.65 (0.47-0.90)	1.06 (0.84-1.35)	0.95 (0.67-1.34)
Prior depressive symptoms^b^	1.18 (1.15-1.20)	1.00 (0.96-1.04)	1.00 (0.97-1.03)
Prior generalized anxiety symptoms^b^	1.03 (0.99-1.08)	1.27 (1.21-1.33)	1.04 (1.00-1.08)
Prior panic symptoms^b^	0.97 (0.94-1.00)	1.01 (0.98-1.05)	1.18 (1.13-1.24)

Abbreviations: ADHD, attention-deficit/hyperactivity symptoms; OR, odds ratio.

^a^ Modeled as a *z* score; coefficients are interpretable as the change in odds of a borderline/clinical level of symptoms per 1-SD increase in level of concern with school violence or shootings.

^b^ Measured before baseline. Variables with no comparison group specified were modeled continuously.

^c^ Includes American Indian or Alaska Native, Native Hawaiian or Pacific Islander, mulitracial, and other race or ethnicity.

### Moderation by Sex and Race/Ethnicity

**Figure 1.**
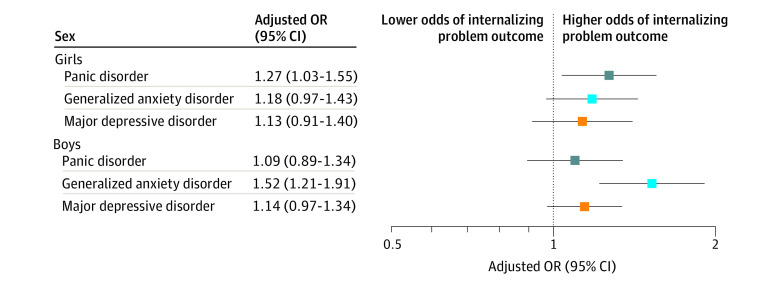
Associations Between Concern About School Violence or Shootings and Surpassing a Borderline/Clinical Symptom Level, Stratified by Sex, for Each Internalizing Problem Outcome Includes 2263 survey respondents. Odds ratios (ORs) are adjusted for age; race and ethnicity; eligibility for free or reduced-cost lunch; delinquent behaviors; attention-deficit/hyperactivity disorder symptoms; past 6-month use of alcohol; past 6-month use of nicotine, marijuana, and/or illicit drugs; past 30-day cyberbullying; subjective social status at school; and continuous scores from prebaseline major depressive disorder, generalized anxiety disorder, and panic disorder.

**Figure 2.**
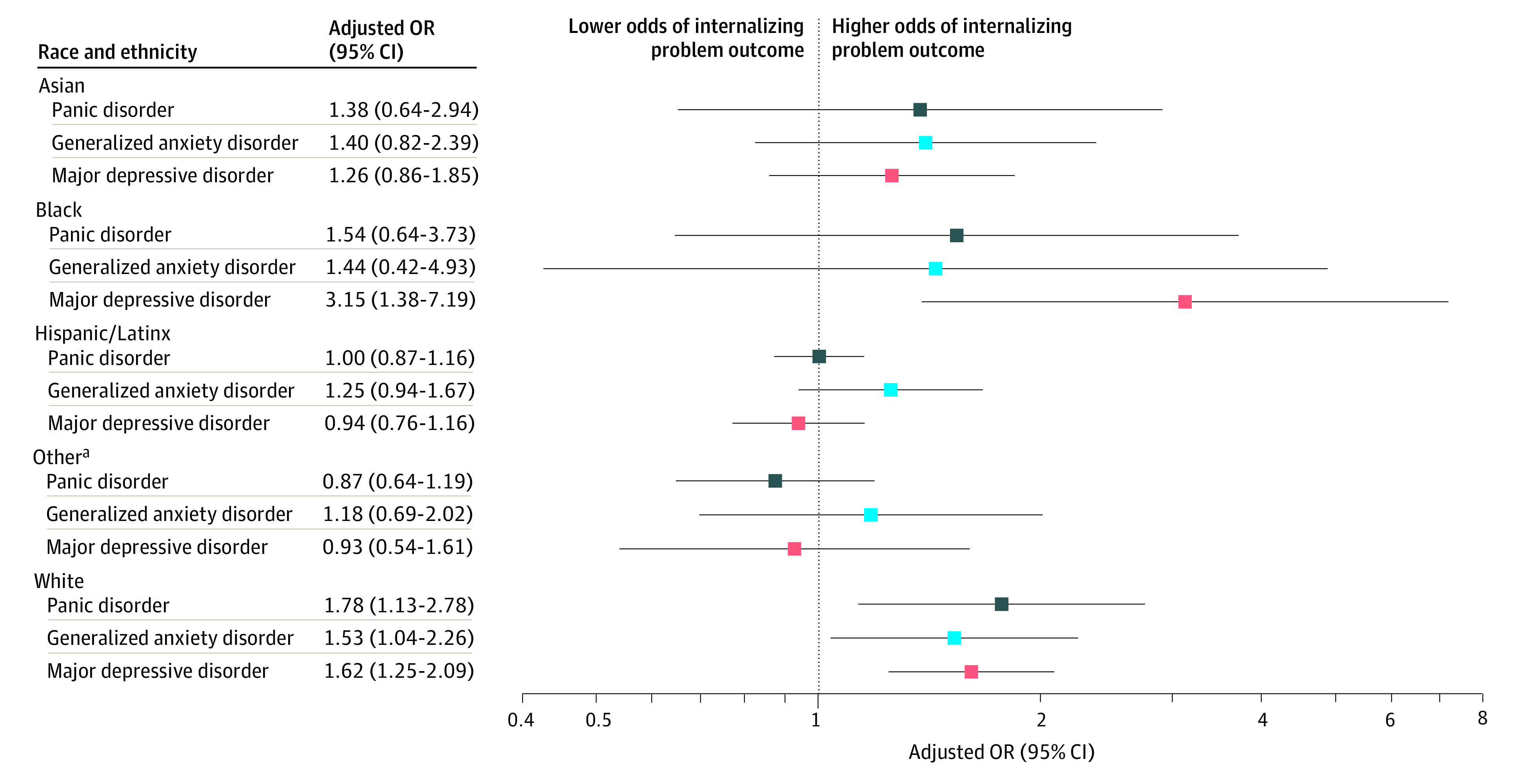
Associations Between Concern About School Violence or Shootings and Surpassing a Borderline/Clinical Symptom Level, Stratified by Race and Ethnicity, for Each Internalizing Problem Outcome Includes 2263 survey respondents. Odds ratios (ORs) are adjusted for age; sex; eligibility for free or reduced-cost lunch; delinquent behaviors; attention-deficit/hyperactivity disorder symptoms; past 6-month use of alcohol; past 6-month use of nicotine, marijuana, and/or illicit drugs; past 30-day cyberbullying; subjective social status at school; and continuous scores from prebaseline major depressive disorder, generalized anxiety disorder, and panic disorder. ^a^Includes American Indian or Alaska Native, Native Hawaiian or Pacific Islander, mulitracial, and other race or ethnicity.

Interaction terms for sex were not significant for any of the internalizing problem outcomes (major depressive disorder, *P* = .93; generalized anxiety disorder, *P* = .055; panic disorder, *P* = .27). Interaction terms for race and ethnicity were significant for major depressive disorder (*P* < .001) and panic disorder (*P* < .001), but not generalized anxiety disorder (*P* = .81). Concern about school violence or shootings was associated with major depressive disorder among Black youth (OR, 3.15; 95% CI, 1.38-7.19) and non-Hispanic/Latinx White youth (OR, 1.62; 95% CI, 1.25-2.09) but not among youth of other races and ethnicities (OR for Asian, 1.26 [95% CI, 0.86-1.85]; OR for Hispanic/Latinx, 0.94 [95% CI, 0.76-1.16]; OR for other, 0.93 [95% CI, 0.54-1.61]). There was an association between concern about school violence or shootings and panic disorder among non-Hispanic/Latinx White youth (OR, 1.78; 95% CI, 1.13-2.78) youth but not among youth of other races and ethnicities. Odds ratios and 95% CIs for surpassing the borderline/clinically significant threshold for major depressive disorder, generalized anxiety disorder, or panic disorder, stratified by sex or race and ethnicity, are shown in Figure 1 and Figure 2, respectively. The sensitivity analyses produced results that were broadly consistent with the main analyses (eMethods and eTables 3 to 5 in the Supplement).

## Discussion

In this prospective study, we found that approximately one-third of adolescents in our sample reported feeling very or extremely concerned, worried, and stressed about shootings and violence at their own school or other schools in 2016. Higher levels of concern about school violence or shootings were associated with heightened odds of meeting borderline/clinical criteria for generalized anxiety disorder and panic disorder 6 months later, even after adjusting for previous level of internalizing problems and 10 additional demographic and externalizing covariates. The strength of some of these associations varied by race and ethnicity; among Black adolescents, the magnitude of the association between concern about school violence or shootings with subsequent major depressive disorder was particularly high compared with the association for adolescents of other races and ethnicities. Against a backdrop of an increase in the prevalence of internalizing problems in US youth^1,2,3^ and concerns over racial and ethnic disparities in youth mental health,^24^ these findings warrant public health attention.

Numerous prior studies have found that exposure to violence in school settings (both direct involvement and witnessing), as well as perceptions of safety at school, are associated with adverse mental health outcomes.^16,25,26,27,28,29^ Our study connects to emerging literature highlighting vicarious effects of school-based violence in that we did not study exposure to violence directly, but rather a concern about violence or shootings both at students’ own schools and at other schools as an overarching societal problem and possible widespread threat. In addition, our results suggest that these concerns could represent a new possible explanation for upward trends in the prevalence of internalizing problems among adolescents, adding to a mounting list of potential causes that includes environmental concerns, digital media use, declining sleep duration, and increasing wealth inequality, among others.^2,3,7^ Together these factors reflect a constellation of social, political, economic, and environmental concerns that may contribute to a perception of “social fracture” among youth.^6^

Unlike other studies,^10,15^ we found no evidence that racial and ethnic groups differed in likelihood of reporting concern about school violence or shootings. However, we did observe a stronger association between these concerns and major depressive disorder among Black students, compared with other students, raising the possibility that concerns with this violence may be a particularly potent risk factor for major depressive disorder among Black youth specifically. Our measure of concern about school violence or shootings did not ask specifically about the source of this violence; for Black youth, the sources of violence in school settings may be more varied than for other youth, including school resource officer programs that place police in schools.^30^ Other scholars have written about how “anticipatory trauma” is a racialized experience that may result in hypervigilant behavior and symptoms of chronic stress, which are common antecedents of internalizing problems.^31,32^ In addition, given the modestly stronger association between concerns about school violence or shootings and panic disorder among non-Hispanic/Latinx White students, an alternative explanation is that youth of different races and ethnicities express internalizing problems through different phenotypes in response to these concerns.

The high proportion of adolescents who reported concern about school violence or shootings may warrant population-based intervention strategies. Tiered, school-based services can provide universal mental health promotion programs to all students, as well as targeted interventions and counseling for students with increased perceived need.^33^ A recent systematic review^34^ suggests, however, that more research is needed on the adaptation of these programs to best meet the needs of students of color. Upstream approaches may also be successful in simultaneously addressing gun violence, perceptions of safety, and internalizing problems. Although more research is needed on long-term efficacy, interventions that include social-emotional learning components and prevent earlier, more minor instances of violence seem especially promising.^28,35^ Finally, beyond the school setting, policies that directly address gun violence (eg, child access prevention laws)^28^ or increase access to mental health services may also be helpful for promoting well-being among youth.

### Limitations

This study has some limitations. The scale measuring concern about school violence or shootings has not been psychometrically validated; however, the items had high internal reliability in this sample. In a related vein, our scale measured concern about shootings and violence at the students’ school and other schools together using a single item stem, and it was not possible to disentangle concerns related to violence vs shootings in particular and at a student’s own vs other schools. The extent to which these results with a regional urban/suburban sample generalize to adolescents across other regions is unknown. We assessed internalizing problems with a self-report inventory of depressive and anxiety symptoms instead of a diagnostic interview. Data collection for this study occurred from the fall of 2015 to fall of 2016, and it is likely that the nature of concerns about school violence has changed since this period, especially given the high school shootings in 2018 in Parkland, Florida, and Santa Fe, Texas, both of which were highly publicized and motivated protests across the country. We included a wide variety of confounders, but other variables of interest, such as neighborhood-level exposure to violence and mental health treatment, were not assessed, and thus residual or unmeasured confounding remains a possibility. We observed some demographic differences between study participants and those not surveyed at baseline, lost to follow-up, or excluded due to missing data, which may affect the generalizability of the results.

## Conclusions

In this longitudinal cohort study, concern about school violence or shootings was associated with anxiety and panic symptoms among adolescents, with variation by race and ethnicity. This study highlights the need for research on interventions that can foster perceptions of safety at schools, prevent downstream violent behaviors, and improve the mental health of youth.

## Notes

Supplement. **eFigure.** Flow Diagram for Participant Selection **eTable 1.** Comparisons Between Adolescents Included and Excluded in the Study Sample **eTable 2.** 2 × 2 Tables Comparing the Proportion of Adolescents Surpassing the Borderline/Clinically Significant Thresholds for Each Internalizing Problem at the Prebaseline and Follow-up Waves **eTable 3.** Adjusted Association of Concern With School Shootings and Violence With Depressive, Generalized Anxiety, and Panic Symptoms, Using Linear Regression and Continuous Symptom Scores as the Outcome (n = 2263) **eTable 4.** Adjusted Association of Concern With School Shootings and Violence With Depressive, Generalized Anxiety, and Panic Symptoms, With the Addition of Depressive Symptoms at the Baseline Wave as a Covariate (n = 2263) **eTable 5.** Adjusted Association of Concern With School Shootings and Violence With Depressive, Generalized Anxiety, and Panic Symptoms, With the Addition of Concerns About Societal Discrimination and Police Brutality as Covariates (n = 2263) **eMethods.** “Issues in Society” Survey Items and Methods and Results of Sensitivity Analyses
