# Supplementary material for: Adolescents’ Concerns About School Violence or Shootings and Association With Depressive, Anxiety, and Panic Symptoms
Source: JAMA Netw Open. 2021 Nov 1;4(11):e2132131. doi: 10.1001/jamanetworkopen.2021.32131 (PMC8561324; doi:10.1001/jamanetworkopen.2021.32131)
Supplement: Supplement. — eFigure. Flow Diagram for Participant Selection eTable 1. Comparisons Between Adolescents Included and Excluded in the Study Sample eTable 2. 2 × 2 Tables Comparing the Proportion of Adolescents Surpassing the Borderline/Clinically Significant Thresholds for Each Internalizing Problem at the Prebaseline and Follow-up Waves eTable 3. Adjusted Association of Concern With School Shootings and Violence With Depressive, Generalized Anxiety, and Panic Symptoms, Using Linear Regression and Continuous Symptom Scores as the Outcome (n = 2263) eTable 4. Adjusted Association of Concern With School Shootings and Violence With Depressive, Generalized Anxiety, and Panic Symptoms, With the Addition of Depressive Symptoms at the Baseline Wave as a Covariate (n = 2263) eTable 5. Adjusted Association of Concern With School Shootings and Violence With Depressive, Generalized Anxiety, and Panic Symptoms, With the Addition of Concerns About Societal Discrimination and Police Brutality as Covariates (n = 2263) eMethods. “Issues in Society” Survey Items and Methods and Results of Sensitivity Analyses [file jamanetwopen-e2132131-s001.pdf]

Supplementary Online Content

Riehm KE, Mojtabai R, Adams LB, et al. Adolescents' concerns about school violence or shootings and association with depressive, anxiety, and panic symptoms. *JAMA Netw Open*. 2021;4(11):e2132131. doi:10.1001/jamanetworkopen.2021.32131

eFigure. Flow Diagram for Participant Selection
eTable 1. Comparisons Between Adolescents Included and Excluded in the Study Sample
eTable 2. 2 × 2 Tables Comparing the Proportion of Adolescents Surpassing the Borderline/Clinically Significant Thresholds for Each Internalizing Problem at the Prebaseline and Follow-up Waves
eTable 3. Adjusted Association of Concern With School Shootings and Violence With Depressive, Generalized Anxiety, and Panic Symptoms, Using Linear Regression and Continuous Symptom Scores as the Outcome (n = 2263)
eTable 4. Adjusted Association of Concern With School Shootings and Violence With Depressive, Generalized Anxiety, and Panic Symptoms, With the Addition of Depressive Symptoms at the Baseline Wave as a Covariate (n = 2263)
eTable 5. Adjusted Association of Concern With School Shootings and Violence With Depressive, Generalized Anxiety, and Panic Symptoms, With the Addition of Concerns About Societal Discrimination and Police Brutality as Covariates (n = 2263)
eMethods. "Issues in Society" Survey Items and Methods and Results of Sensitivity Analyses

This supplementary material has been provided by the authors to give readers additional information about their work.

© 2021 Riehm KE et al. *JAMA Network Open*.

eFigure. Flow Diagram for Participant Selection

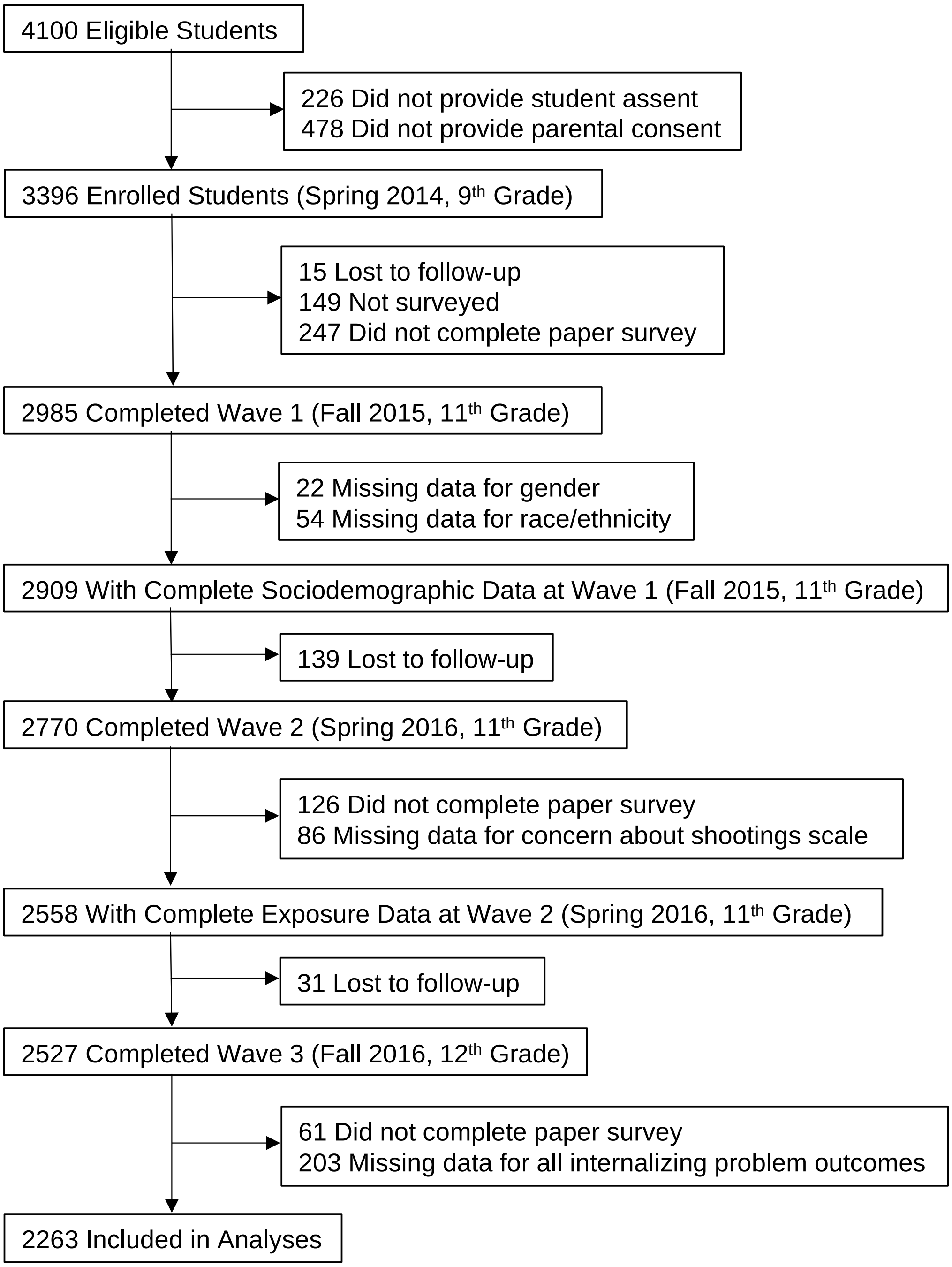

© 2021 Riehm KE et al. *JAMA Network Open*.

eTable 1. Comparisons Between Adolescents Included and Excluded in the Study Sample

Variables	N (%) In Analytic Sample (n=2,263)	N (%) Among Those Excluded from Analyses (n=1,133)	P-value for Comparison[b]
Concern with School Shootings and Violence[a]	1.58 (1.27)	1.34 (1.33)	<0.01
Age[a]	16.47 (0.39)	16.60 (0.49)	<0.01
Gender			
Female	1,250 (55.2%)	551 (49.8%)	<0.01
Male	1,013 (44.8%)	555 (50.2%)	
Ethnicity			
White	375 (16.6%)	145 (13.8%)	<0.01
Black	89 (3.9%)	77 (7.3%)	
Hispanic/Latino	1,001 (44.2%)	556 (53.1%)	
Asian	444 (19.6%)	91 (8.7%)	
Other	354 (15.6%)	179 (17.1%)	
Eligibility for reduced cost or free lunch			
Not eligible	1,148 (53.6%)	185 (43.1%)	<0.01
Free or reduced cost	992 (46.4%)	244 (56.9%)	

Notes: Estimates are based on available data. [a]Reported as mean (SD). [b]P-values were calculated using t-tests for continuous variables and chi-squared tests for categorical variables.
Excluded due to not being surveyed at baseline, non-completion of the paper form of the survey (which included the concern with school shooting and violence items), loss to follow-up, and/or missing exposure/outcome data.

© 2021 Riehm KE et al. *JAMA Network Open*.

eTable 2. 2 × 2 Tables Comparing the Proportion of Adolescents Surpassing the Borderline/Clinically Significant Thresholds for Each Internalizing Problem at the Prebaseline and Follow-up Waves

**Internalizing Problem (Baseline)**	**Follow-Up, n (%)**	
	**No**	**Yes**
Depressive Symptoms		
No	1576 (91.3)	150 (8.7)
Yes	178 (50.1)	177 (49.9)
Generalized Anxiety Symptoms		
No	1650 (92.0)	143 (8.0)
Yes	169 (58.5)	120 (41.5)
Panic Symptoms		
No	1644 (92.2)	140 (7.8)
Yes	141 (49.0)	147 (51.0)
Note: Based on available data. Percentages sum across columns.		

© 2021 Riehm KE et al. *JAMA Network Open*.

eTable 3. Adjusted Association of Concern With School Shootings and Violence With Depressive, Generalized Anxiety, and Panic Symptoms, Using Linear Regression and Continuous Symptom Scores as the Outcome (n = 2263)

**Variable**	**Outcome**					
	**Depressive Symptoms**		**Generalized Anxiety Symptoms**		**Panic Symptoms**	
	**β**	**95% CI**	**β**	**95% CI**	**β**	**95% CI**
**Adjusted Model**						
Concern with School Shootings and Violence[a]	**0.25**	**0.06, 0.43**	**0.40**	**0.26, 0.55**	**0.37**	**0.22, 0.52**
Sex[b]						
Female	ref.	ref.	ref.	ref.	ref.	ref.
Male	**-0.99**	**-1.32, -0.66**	**-0.83**	**-1.01, -0.64**	**-1.03**	**-1.30, -0.76**
Age	0.08	-0.32, 0.47	-0.23	-0.66, 0.19	0.13	-0.32, 0.58
Race/Ethnicity						
White	ref.	ref.	ref.	ref.	ref.	ref.
Black	**1.10**	**0.09, 2.12**	-0.15	-0.81, 0.51	0.03	-0.85, 0.90
Hispanic/Latinx	-0.14	-0.78, 0.49	0.07	-0.48, 0.63	-0.34	-0.79, 0.11
Asian	0.33	-0.41, 1.08	0.03	-0.58, 0.63	-0.31	-0.95, 0.33
Other	0.48	-0.42, 1.39	0.23	-0.32, 0.79	-0.07	-0.68, 0.54
Free or Reduced Cost Lunch						
Not Eligible	ref.	ref.	ref.	ref.	ref.	ref.
Eligible	0.09	-0.54, 0.73	-0.24	-0.64, 0.15	0.01	-0.53, 0.54
Cyberbullying						
Not Victimized	ref.	ref.	ref.	ref.	ref.	ref.
Victimized	0.28	-0.74, 1.29	-0.46	-1.02, 0.10	0.23	-0.34, 0.80
Subjective Social Status at School	**-0.21**	**-0.33, -0.08**	-0.04	-0.15, 0.06	-0.04	-0.13, 0.04
Delinquent Behavior	0.01	-0.07, 0.08	-0.01	-0.04, 0.02	**-0.06**	**-0.12, -0.01**
ADHD Symptoms	**0.10**	**0.07, 0.13**	**0.06**	**0.04, 0.08**	**0.08**	**0.05, 0.11**
Alcohol Use						
No	ref.	ref.	ref.	ref.	ref.	ref.
Yes	0.00	-0.33, 0.34	0.05	-0.18, 0.28	-0.08	-0.51, 0.35
Nicotine, Cannabis and Drug Use						
No	ref.	ref.	ref.	ref.	ref.	ref.
Yes	0.45	-0.08, 0.98	0.10	-0.28, 0.47	0.08	-0.24, 0.40
Prior Depressive Symptoms	**0.49**	**0.44, 0.54**	0.01	-0.04, 0.07	-0.02	-0.06, 0.03
Prior Generalized Anxiety Symptoms	**0.10**	**0.04, 0.15**	**0.49**	**0.42, 0.55**	**0.07**	**0.03, 0.11**
Prior Panic Symptoms	-0.04	-0.12, 0.04	0.02	-0.04, 0.08	**0.42**	**0.33, 0.52**

Notes: [a]Modelled as a z-score; coefficients are interpretable as the change in symptom score per one standard deviation increase in level of concern with school shootings and violence. [b]Coefficients for sex are not directly comparable to those in the main analyses because the symptom cutoffs are sex-specific and require a lower symptom level to surpass the threshold for males than for females.

© 2021 Riehm KE et al. *JAMA Network Open*.

eTable 4. Adjusted Association of Concern With School Shootings and Violence With Depressive, Generalized Anxiety, and Panic Symptoms, With the Addition of Depressive Symptoms at the Baseline Wave as a Covariate (n = 2263)

**Variable**	**Outcome**					
	**Depressive Symptoms**		**Generalized Anxiety Symptoms**		**Panic Symptoms**	
	**OR**	**95% CI**	**OR**	**95% CI**	**OR**	**95% CI**
Concern with School Shootings and Violence[a]	1.09	0.95, 1.25	**1.28**	**1.11, 1.48**	**1.15**	**1.01, 1.31**
Sex						
Female	ref.	ref.	ref.	ref.	ref.	ref.
Male	**1.82**	**1.44, 2.30**	**1.81**	**1.34, 2.45**	**3.02**	**2.11, 4.32**
Age	1.19	0.81, 1.74	0.79	0.59, 1.04	1.13	0.69, 1.85
Race/Ethnicity						
White	ref.	ref.	ref.	ref.	ref.	ref.
Black	1.13	0.76, 1.70	1.01	0.49, 2.07	1.22	0.64, 2.36
Hispanic/Latinx	1.14	0.86, 1.53	1.46	0.93, 2.29	0.79	0.54, 1.15
Asian	1.33	0.91, 1.93	1.15	0.49, 2.69	1.00	0.54, 1.84
Other	1.31	0.78, 2.18	1.19	0.56, 2.50	0.87	0.66, 1.16
Free or Reduced Cost Lunch						
Not Eligible	ref.	ref.	ref.	ref.	ref.	ref.
Eligible	1.22	0.85, 1.75	0.95	0.72, 1.25	1.20	0.85, 1.69
Cyberbullying						
Not Victimized	ref.	ref.	ref.	ref.	ref.	ref.
Victimized	1.06	0.69, 1.63	1.00	0.61, 1.62	1.23	0.86, 1.74
Subjective Social Status at School	0.97	0.89, 1.06	1.02	0.92, 1.14	1.07	0.97, 1.17
Delinquent Behavior	1.00	0.97, 1.02	**1.02**	**1.01, 1.03**	0.97	0.93, 1.01
ADHD Symptoms	**1.03**	**1.01, 1.04**	1.01	1.00, 1.03	**1.03**	**1.02, 1.05**
Alcohol Use						
No	ref.	ref.	ref.	ref.	ref.	ref.
Yes	1.01	0.65, 1.57	0.94	0.65, 1.36	0.90	0.68, 1.18
Nicotine, Cannabis and Drug Use						
No	ref.	ref.	ref.	ref.	ref.	ref.
Yes	**1.44**	**1.04, 1.99**	0.93	0.74, 1.16	1.00	0.70, 1.44
Prior Depressive Symptoms	**1.13**	**1.11, 1.16**	0.96	0.92, 1.00	**0.96**	**0.94, 0.99**
Prior Generalized Anxiety Symptoms	1.02	0.98, 1.07	**1.27**	**1.20, 1.34**	1.03	0.98, 1.08
Prior Panic Symptoms	**0.97**	**0.94, 0.99**	1.01	0.98, 1.05	**1.19**	**1.14, 1.24**
Baseline Depressive Symptoms[b]	**1.06**	**1.05, 1.06**	**1.04**	**1.03, 1.05**	**1.05**	**1.04, 1.06**

Notes: [a]Modelled as a z-score; coefficients are interpretable as the change in symptom score per one standard deviation increase in level of concern with school shootings and violence. [b]Measured with the Center for Epidemiologic Studies Depression Scale (modelled as a continuous score with range 0-60).

© 2021 Riehm KE et al. *JAMA Network Open*.

eTable 5. Adjusted Association of Concern With School Shootings and Violence With Depressive, Generalized Anxiety, and Panic Symptoms, With the Addition of Concerns About Societal Discrimination and Police Brutality as Covariates (n = 2263)

**Variable**	**Outcome**					
	**Depressive Symptoms**		**Generalized Anxiety Symptoms**		**Panic Symptoms**	
	**OR**	**95% CI**	**OR**	**95% CI**	**OR**	**95% CI**
Concern with School Shootings and Violence[a]	1.07	0.88, 1.3	**1.23**	**1.04, 1.46**	1.13	0.98, 1.31
Sex						
Female	ref.	ref.	ref.	ref.	ref.	ref.
Male	**1.61**	**1.30, 1.98**	**1.67**	**1.25, 2.22**	**2.71**	**1.96, 3.76**
Age	1.16	0.80, 1.68	0.79	0.60, 1.04	1.12	0.68, 1.84
Race/Ethnicity						
White	ref.	ref.	ref.	ref.	ref.	ref.
Black	1.20	0.78, 1.84	1.06	0.46, 2.42	1.28	0.64, 2.57
Hispanic/Latinx	1.06	0.79, 1.43	1.39	0.88, 2.20	0.77	0.49, 1.20
Asian	1.29	0.91, 1.85	1.12	0.48, 2.62	1.01	0.55, 1.83
Other	1.42	0.90, 2.26	1.26	0.59, 2.69	0.96	0.69, 1.34
Free or Reduced Cost Lunch						
Not Eligible	ref.	ref.	ref.	ref.	ref.	ref.
Eligible	1.26	0.88, 1.79	0.99	0.75, 1.31	1.25	0.87, 1.80
Cyberbullying						
Not Victimized	ref.	ref.	ref.	ref.	ref.	ref.
Victimized	1.15	0.76, 1.75	1.07	0.67, 1.72	1.32	0.95, 1.85
Subjective Social Status at School	0.93	0.86, 1.01	0.99	0.88, 1.11	1.02	0.93, 1.12
Delinquent Behavior	1.00	0.96, 1.04	**1.02**	**1.01, 1.04**	0.97	0.92, 1.02
ADHD Symptoms	**1.04**	**1.02, 1.05**	**1.02**	**1.00, 1.04**	**1.04**	**1.03, 1.06**
Alcohol Use						
No	ref.	ref.	ref.	ref.	ref.	ref.
Yes	0.97	0.65, 1.47	0.93	0.66, 1.32	0.91	0.66, 1.24
Nicotine, Cannabis and Drug Use						
No	ref.	ref.	ref.	ref.	ref.	ref.
Yes	**1.55**	**1.10, 2.19**	0.95	0.73, 1.24	1.06	0.77, 1.48
Prior Depressive Symptoms	**1.17**	**1.15, 1.20**	1.00	0.96, 1.03	1.00	0.97, 1.04
Prior Generalized Anxiety Symptoms	1.03	0.99, 1.07	**1.27**	**1.21, 1.33**	1.03	0.99, 1.08
Prior Panic Symptoms	**0.97**	**0.95, 0.99**	1.01	0.98, 1.05	**1.18**	**1.13, 1.24**
Concern with Police Brutality[b]	0.97	0.85, 1.10	0.98	0.84, 1.15	0.96	0.83, 1.12
Concern with Societal Discrimination[b]	**1.16**	**1.02, 1.31**	**1.15**	**1.00, 1.32**	1.13	0.94, 1.36

Notes: [a]Modelled as a z-score; coefficients are interpretable as the change in symptom score per one standard deviation increase in level of concern with school shootings and violence. [b]These scales were scored in the same way as the concern with school shootings and violence scale (range of 0-4, with higher scores indicating greater concern).

© 2021 Riehm KE et al. *JAMA Network Open*.

eMethods. "Issues in Society" Survey Items and Methods and Results of Sensitivity Analyses

<u>"Issues in Society" survey items</u>

1. **Please rate your degree of *concern, worry,* and *stress* towards the following issues in terms of *their effect on you personally.***

a. Increasing hostility and discrimination of people because of their race, ethnicity, sexual orientation/identity, immigrant status, religion, or disability status in society.

Concerned W6_Concern_Soc_1	***Worried*** W6_Concern_Soc_2	***Stressed*** W6_Concern_Soc_3
O Not at all	O Not at all	O Not at all
O Slightly	O Slightly	O Slightly
O Somewhat	O Somewhat	O Somewhat
O Very	O Very	O Very
O Extremely	O Extremely	O Extremely

b. Shootings or violence at your school or other schools.

Concerned W6_Concern_Shoot_1	***Worried*** W6_Concern_Shoot_2	***Stressed*** W6_Concern_Shoot_3
O Not at all	O Not at all	O Not at all
O Slightly	O Slightly	O Slightly
O Somewhat	O Somewhat	O Somewhat
O Very	O Very	O Very
O Extremely	O Extremely	O Extremely

c. Police brutality or the unfair treatment of members in your community by law enforcement.

Concerned W6_Concern_Law_1	***Worried*** W6_Concern_Law_2	***Stressed*** W6_Concern_Law_3
O Not at all	O Not at all	O Not at all
O Slightly	O Slightly	O Slightly
O Somewhat	O Somewhat	O Somewhat
O Very	O Very	O Very
O Extremely	O Extremely	O Extremely

<u>Methods and results of sensitivity analyses</u>

We conducted three sets of sensitivity analyses to test the sensitivity of our results to modelling decisions and potential confounding.

1. In our main analyses, we dichotomized scores from the RCADS for depressive, generalized anxiety, and panic symptoms. To test sensitivity of our results to this decision, we re-estimated the adjusted models using linear regression with continuous symptom scores as outcomes. These analyses are displayed in eTable 2. Concern about school shootings and violence was associated with all three internalizing problem outcomes, suggesting that these associations are present at different levels of symptom severity and not limited to borderline/clinical levels.
2. It is possible that some students who did not report internalizing symptoms at the time of the pre-baseline wave may have developed them by the time of the baseline wave. To test sensitivity of our results to this possibility, we included depressive symptoms measured by the Center for Epidemiologic Studies Depression Scale as an additional covariate (the RCADS was not administered at the baseline wave). These analyses are displayed in eTable 3. Results were broadly consistent with the main analyses.

© 2021 Riehm KE et al. *JAMA Network Open*.

3. It is possible that other concerns about society may also be associated with internalizing problems among adolescents and could confound the associations observed in our study. In the Happiness and Health survey, two other concerns were measured at the baseline wave: concern with increasing societal discrimination and concern with police brutality. These concerns were measured in the same manner as the concern with school shooting and violence variable; we re-estimated the adjusted model and included these as additional covariates. These analyses are displayed in eTable 4. Associations between concern with school shootings and violence and each internalizing problem outcome were attenuated slightly, but the pattern of results was otherwise broadly consistent with the main analyses.

© 2021 Riehm KE et al. *JAMA Network Open*.
